# Ischemic Bilateral Opercular Syndrome

**DOI:** 10.1155/2013/513572

**Published:** 2013-02-18

**Authors:** Aysel Milanlioglu, Mehmet Nuri Aydın, Alper Gökgül, Mehmet Hamamcı, Mehmet Atilla Erkuzu, Temel Tombul

**Affiliations:** Neurology Department, Faculty of Medicine, Yüzüncü Yıl University, Turkey

## Abstract

Opercular syndrome, also known as Foix-Chavany-Marie syndrome, is a paralysis of the facial, pharyngeal, masticatory, tongue, laryngeal, and brachial muscles. It is a rare cortical form of pseudobulbar palsies caused by vascular insults to bilateral operculum. Its clinical presentations include anarthria, weakness of voluntary muscles involving face, tongue, pharynx, larynx, and masticatory muscles. However, autonomic reflexes and emotional activities of these structures are preserved. In the present case, an 81-year-old male presented with acute onset of anarthria with difficulties in chewing, speaking, and swallowing that was diagnosed with opercular syndrome.

## 1. Introduction 

Opercular syndrome (OPS) is a rarely seen cortical type of pseudobulbar palsy that is characterized by paralysis of facial, lingual, pharyngeal, and masticatory voluntary muscles with preservation of autonomic, involuntary, and reflexive functions [1]. OPS, facio-labio-glosso-pharyngo- laryngo-brachial paralysis, was first described by French physicians, Foix, in 1926 [2]. However, the first case was reported by Magnus (from Germany) in 1837 [3]. The lesions are usually located at the anterior part of the operculum, so it is also called anterior OPS.

Herein, we report an 81-year-old male presenting with acute onset of aphonia, and facio-labio-glosso-pharyngo-laryngeal paralysis with preservation of involuntary and emotional activities. 

## 2. Case Report 

An 81-year-old right-handed male presented with acute onset of sound volume loss, inability to swallow and speak, inability to move tongue, and difficulties in chewing the foods. The past medical history was remarkable for diabetes mellitus, hypertension, coronary artery disease, and benign prostate hypertrophy. His first cerebral vascular event presenting with right-sided arm and leg weakness was observed about 4 years ago and his weakness was recovered completely. However, he has not used suggested antiplatelet treatment up to now. The family history was unremarkable. 

**Figure 1:**
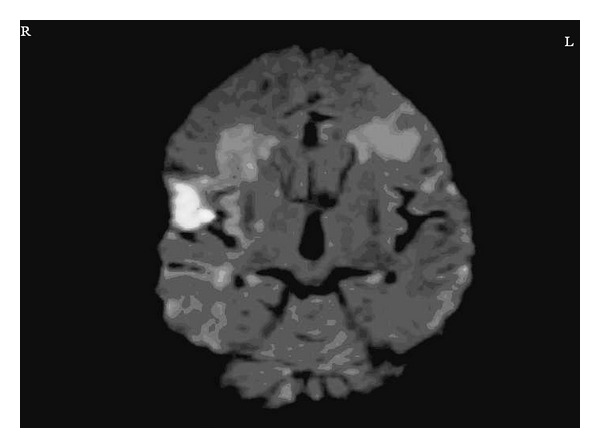
Diffusion-weighted magnetic resonance imaging demonstrates right frontal opercular restricted diffusion which is diagnosed as acute opercular infarct (R: Right, L: Left).

**Figure 2:**
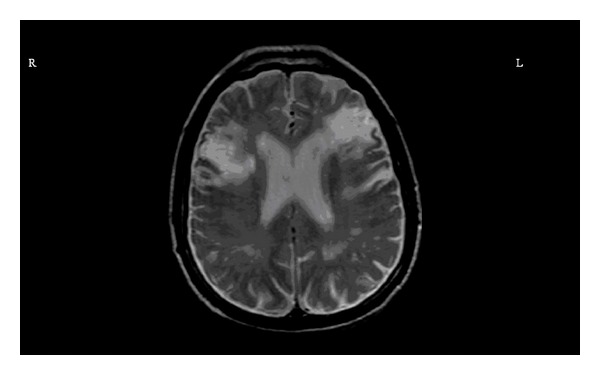
Magnetic resonance imaging sequences demonstrate bilateral inferior frontal gyrus chronic infarct (R: Right, L: Left).

In neurological assessment, he was aphonic, but his verbal and reading perception was normal. He was able to answer the questions with signs and writing. The pupillary, corneal reflexes, and extraocular movements were intact. The left nasolabial sulcus disappeared. He was not able to open the mouth, protrude the tongue, show the teeth, whistle, chew, and swallow. The tongue was on the midline and immobile. No tongue atrophy, fibrillation, and deviation were observed. The taste sensation was intact. Palatal, laryngeal, blink, and gag reflexes, spontaneous smiling, and yawning were protected. The upper and lower limbs muscles strengths were 5/5. Deep tendon reflex was normoactive. Babinski response was absent. Extrapyramidal, coordination, and sensory examinations were normal. No vocal cord paralysis was detected on the laryngoscopic examination. The laboratory investigation including liver and kidney function, complete cell count, urinalysis, and erythrocyte sedimentation rate was normal. A transthoracic echocardiogram showed biatrial dilatation, mild calcification of mitral and aortic valves, and mild aortic and mitral valves insufficiency. A doppler ultrasonography of carotid and vertebral arteries was unremarkable. The magnetic resonance imaging (MRI) of brain indicated right frontal opercular acute infarct with 3.3 × 2.6 cm on dimensions (Figure 1) and bilateral chronic inferior frontal gyrus infarct (Figure 2). He was treated with 300 mg acetylsalicylic acid daily and was placed nasogastric tube for feeding due to lack of swallowing function. At the end of the 3rd week, he had nonsense sounds and was able to open the mouth. However, there was no improvement in speech, chewing, and swallowing functions. 

## 3. Discussion 

OPS is characterized by anarthria and paralysis of the facial, pharyngeal, masticatory, tongue, laryngeal, and brachial voluntary muscles. Operculum refers to the brain cortex covering the insula, inferior frontal, pre- and postcentral, supramarginal, angular (inferior parietal), and superior temporal gyrus. OPS is particularly originated from the damages that are located in posterior part of the inferior frontal gyrus and inferior part of precentral gyrus [4]. There are several connections between bilateral precentral gyrus and cranial nerves 5, 6, 9, 10, and 12, and bilateral damage of these corticobulbar tracts can cause OPS. 

The voluntary muscle controls of the face, tongue, and pharynx are provided by primary motor cortex and pyramidal tract, whereas the spontaneous and emotional controls are provided by thalamus, hypothalamus, and extrapyramidal tract [5]. The selective paralysis of voluntary muscle weakness in OPS is named as “autonomic-voluntary dissociation.” In our case, the typical autonomic-voluntary dissociation was observed.

The etiologic factors of OPS include thrombotic or embolic multiple strokes, head trauma, tumor, developmental perisylvian dysplasia, multiple sclerosis, acute disseminated encephalomyelitis, moyamoya disease, vasculitis, and neurodegenerative disease [6]. Ischemic stroke related OPS is the most common classic type. In this type of OPS, as seen in our case, patients usually have history of one or more contralateral stroke. Unilateral lesion rarely can cause OPS, but it was postulated that those cases might have subtle contralateral lesions. The SPECT imaging study on the patients with unilateral OPS showed cerebral diaschisis that supported the hypothesis [7]. We think that combination of both old left inferior frontal gyrus lesion and the new acute infarct of the operculum contributed to the clinical picture of OPS. 

Pseudobulbar paralysis in OPS is clinically distinguished from bulbar paralysis, disorders of the cranial nerves and neuromuscular junction (e.g., botulism and myasthenia gravis) by normal eye movements, preserved or hyperactive brainstem reflexes (e.g., jaw jerk), the dissociation of automatic and volitional movements of the bulbar muscles with preservation of automatic movements, and the absence of atrophy and fasciculations of the lower motor neuron-innervated muscles [8].

The treatment and prognosis of OPB is related to underlying etiological factors. However, clinical improvement is usually poor. Chewing, swallowing, and speech functions do not usually recover completely. Patients with OPS have a significant risk for aspiration pneumonia. Therefore, during acute treatment and rehabilitation process, speech dysfunction and feeding are two most important issues. In our case, no improvement in speech, swallowing, and chewing functions was observed. He could produce nonsense sounds and open the mouth at the end of the 3rd weeks. Preserving patients from OPS can be possible via preventive treatment of repetitive stroke.

## References

[1] Ann MY, Liu OK, Wu YL (2001). Foix-chavany-marie syndrome. *Chinese Medical Journal*.

[2] Foix C, Chavany JA, Marie J (1926). Diplegie facio-linguo-masticatrice d'origine cortico-sous-cortical sans paralysie des members. *Revue Neurologique*.

[3] Magnus A (1837). Fall von aufhebung des willenseinflusses auf einige hirnnerven. *Archiv für Anatomie, Physiologie und Wissenschaftliche Medicin*.

[4] Weller M (1993). Anterior opercular cortex lesions cause dissociated lower cranial nerve palsies and anarthria but no aphasia: foix-chavany-marie syndrome and “automatic voluntary dissociation” revisited. *Journal of Neurology*.

[5] Mao CC, Coull BM, Golper LAC, Rau MT (1989). Anterior operculum syndrome. *Neurology*.

[6] Bakar M, Kirshner HS, Niaz F (1998). The opercular-subopercular syndrome: four cases with review of the literature. *Behavioural Neurology*.

[7] Kutluay E, Colakoglu Z, Dirlik A, Kumral K (1996). Brain SPECT in anterior opercular syndrome due to a unilateral lesion. *Journal of Neurology*.

[8] Lanska DJ Foix-chavany-marie syndrome. http://www.Medlink.com/medlinkcontent.asp.

